# Sepsis-Induced Brain Dysfunction: Pathogenesis, Diagnosis, and Treatment

**DOI:** 10.1155/2022/1328729

**Published:** 2022-08-24

**Authors:** Shangwen Pan, Zheng Lv, Rui Wang, Huaqing Shu, Shiying Yuan, Yuan Yu, You Shang

**Affiliations:** Department of Critical Care Medicine, Union Hospital, Tongji Medical College, Huazhong University of Science and Technology, Wuhan, China

## Abstract

Dysregulated host response to infection, which cause life-threatening organ dysfunction, was defined as sepsis. Sepsis can cause acute and long-term brain dysfunction, namely, sepsis-associated encephalopathy (SAE) and cognitive impairment. SAE refers to changes in consciousness without direct evidence of central nervous system infection. It is highly prevalent and may cause poor outcomes in sepsis patients. Cognitive impairment seriously affects the life quality of sepsis patients and increases the medical burden. The pathogenesis of sepsis-induced brain dysfunction is mainly characterized by the interaction of systemic inflammation, blood-brain barrier (BBB) dysfunction, neuroinflammation, microcirculation dysfunction, and brain dysfunction. Currently, the diagnosis of sepsis-induced brain dysfunction is based on clinical manifestation of altered consciousness along with neuropathological examination, and the treatment is mainly involves controlling sepsis. Although treatments for sepsis-induced brain dysfunction have been tested in animals, clinical treat sepsis-induced brain dysfunction is still difficult. Therefore, we review the underlying mechanisms of sepsis-induced brain injury, which mainly focus on the influence of systemic inflammation on BBB, neuroinflammation, brain microcirculation, and the brain function, which want to bring new mechanism-based directions for future basic and clinical research aimed at preventing or ameliorating brain dysfunction.

## 1. Introduction

Sepsis is a life-threatening organ dysfunction syndrome caused by the host's maladjusted responses to infection [1]. Globally, there are >30 million sepsis patients each year [2]. Organ dysfunction is a major complication of sepsis, and sepsis-induced brain dysfunction is highly prevalent and has an early onset [3]. Brain dysfunction is mainly caused by various factors released during sepsis, and clinical examinations have not uncovered any evidence of direct central nervous system (CNS) infection. At acute phase, sepsis-induced brain dysfunction is manifested as sepsis-associated encephalopathy (SAE), delirium, sickness behavior, and cerebral ischemia and hemorrhage, which all associated with cognitive impairment [4]. At long-term phase, cognitive impairment is the main feature of sepsis-induced brain dysfunction [5].

SAE is a main manifestation of sepsis, which is characterized by changes in consciousness that range from confusion to delirium or even coma [6] and affects up to 70% of patients with sepsis [7]. The occurrence of SAE often increases the stay in intensive care unit (ICU) and mortality among sepsis patients [8]. Advancements in medical technology have significantly improved the survival rate of sepsis patients but increased the incidence of long-term sequelae and cognitive impairment, which is as high as 21% [9]. Although such huge challenges stand before us, effective diagnostic strategies and intervention measures in sepsis-induced brain dysfunction, especially SAE and cognitive impairment, are lacking [10]. Currently, only daily neurological examination combined with relevant laboratory examinations can be used to exclusively diagnose SAE, and cognitive instruments were used to diagnose cognitive impairment. Thus, effective techniques for early diagnosis and treatment and even reversal of SAE and cognitive impairment are urgently needed. Here, we review current knowledge on SAE and cognitive impairment and highlight potential diagnostic and treatment strategies.

## 2. Pathogenesis

**Figure 1:**
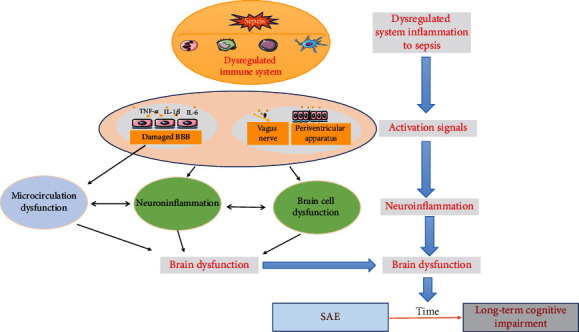
Pathophysiology of sepsis-induced brain dysfunction. Sepsis causes inflammation response, which induces neuroinflammation, microcirculation turbulence, and brain dysfunction.

Sepsis can cause brain damage through a variety of mechanisms. Among them systemic inflammation, BBB dysfunction, neuroinflammation, microcirculation dysfunction, and brain dysfunction were studied well. Sepsis can amplify adverse reactions through the interaction of these mechanisms, which at last resulting in white matter damage and cerebral dysfunction (Figure 1).

### 2.1. System Inflammation

Dysregulation of system inflammatory response is the most important feature of sepsis, which stands throughout the whole process [11]. At acute phase, damage-associated molecular patterns (DAMPs) or pathogen-associated molecular patterns (PAMPs) could be detected by pattern-recognition receptors (PRRs) in immune cells, which at last induce cytokine storm and activate immune system [11]. Currently, the most studied PRRs includes Toll-like receptors (TLRs), nucleotide-binding oligomerization domain- (NOD-) like receptors, retinoic acid-inducible gene- (RIG-) like receptors, mannose-binding lectin, and scavenger receptors. Cytokines including interleukin- (IL-) 1, IL-6, tumor necrosis factor-*α* (TNF-*α*), interferon (IFN) regulatory factor 7 (IRF7), and adaptor protein 1 (AP-1) are produced after activating of PRRs during sepsis [12]. Furthermore, sepsis also induces the activation of inflammasome, which promotes the release of cytokines IL-1*β* and IL-18 [13]. Interestingly, a new form of programmed cell death, named “pyroptosis,” was suggested to involve in sepsis, which can not only lead to direct cell destruction but also to an inflammatory cascade in sepsis [14, 15]. The mechanisms of pyroptosis were mainly include the canonical caspase-1-dependent pathway and the noncanonical caspase-4/5/11-dependent pathway [14]. In canonical pathway, intracellular PRRs recognize stimuli and cleave procaspase-1 into caspase-1, which then promotes the formation of GSDMD channel and the release of IL-1*β*and IL-18 [14]. In noncanonical pathway, HMGB-1 derived from liver cells could promote lipopolysaccharide (LPS) transport into cytoplasm through receptor for advanced glycation end products (RAGE) on vascular endothelial cells and macrophages, which cause the activation of caspase-11 and the formation of GSDMD channel [16]. At the same time, the activated caspase-11 could promote the secretion of IL-1*β* and IL-18 through pannexin-1/P2X7/NLRP3 pathway [17]. Recently, caspase-3 and caspase-8 were suggested to mediate pyroptosis. After activated, caspase-3 induces cell pyroptosis and releases proinflammatory cell mediators through cleaved and activated gasdermin E (GSDME) to form GSDME channel [18]. The activated caspase-8 by blocked transforming growth factor *β*-activated kinase 1 (TAK1) could mediate pyroptosis and inflammatory response in a manner of GSDMD channel [19]. In a word, pyrotosis maybe a reason and a potential therapeutic target for multiple organ dysfunction in sepsis [15]. All immune cells of the innate immune system are mobilized to participate in the process of sepsis [20]. Neutrophils migrate to the inflammation site, where they perform anti-inflammation and eliminate pathogens [20]. The activated mononuclear/macrophage cells phagocytose, kill pathogens, and present antigens [21]. Effector T cells could cause damage by promoting macrophage activation [22]. Abnormal monocyte metabolism is associated with immunosuppression [23].

With the progression of sepsis, multiple organ failure appears, among which the central nervous system is one of the most dangerous organs [24]. Systemic inflammation caused by sepsis not only affects the acute stage of sepsis-induced brain injury, namely, SAE, but also is closely associated with long-term cognitive impairment in the long-term stage of sepsis [25], suggesting that systemic inflammatory response is not only an important influence factor of sepsis-induced brain injury, but also an important intervention target.

### 2.2. Changes in the Blood-Brain Barrier (BBB)

The increased permeability of BBB during sepsis is increasingly accepted, because vasogenic edema and white matter hyperintensities were presented on MRI in SAE patients, indicating BBB disruption [26]. Although sepsis cause BBB damage is not entirely elucidated, several mechanisms have been postulated. The BBB is primarily comprised of microvascular endothelial cells (ECs), tight junction (TJ) proteins, astrocyte endfeet, pericytes, and capillary basement membrane, which are adversely affected by sepsis [27–30]. In normal physiologic conditions, the BBB serves as a physical barrier because TJ between adjacent ECs restrict molecules from diffusing through ECs, ensuring that most molecular trafficking takes a controlled transcellular route across the BBB. In sepsis patients, the expression of TJ was reduced in the brain tissue, indicating BBB damage [27]. Sepsis could activate Toll-like receptor 4/nuclear factor-k-gene binding (TLR4/NF-kB) pathway to change the structure and function of TJ [31]. Histones released during sepsis could induced TJ disruption [32]. Another way is imbalance the oxidative and antioxidant responses of ECs by inducing reactive oxygen species (ROS), which ultimately damage TJ [33]. Inhibiting endothelial nitric oxide synthase (eNOS) and Guanosine triphosphate cyclohydrolase 1 (GTPCH1) and increasing the activation of caspase-3/7 at last promote EC apoptosis during sepsis [29]. Furthermore, DAPMs that are released during sepsis such as ATP could induce apoptosis by purinergic receptor (P2X7R) in brain ECs [28]. Microglia, astrocytes, pericytes, and neutrophils participate in damaging the BBB in sepsis through inflammatory cascade amplification. Besides, astrocytes could expression of vascular endothelial growth factor A (VEGF-A), followed by activating eNOS, inhibiting the expression of claudin-5, and occluding [34]. Collectively, all these factors are ultimately disrupting the barrier function of the BBB. The integrity of basement membrane is damaged in sepsis. The loss of BBB permeability and integrity is a major cause of sepsis-induced brain dysfunction and resultant systemic damage.

ECs activation plays an important role in BBB integrity and is the earliest event in CNS inflammation upon sepsis onset [35]. Activated ECs express various adhesion molecules, including tumor necrosis factor receptor superfamily member 5 (CD40), e-selectin, vascular cell adhesion molecule (VCAM), intercellular cell adhesion molecule (ICAM), and inflammatory receptors, including IL-1, TNF-*α*, and TLR4, which facilitate the entrance of leukocytes and inflammatory mediator into the brain parenchyma [30, 36]. Activation of the EC I*κ*B-*α*/NF-*κ*B signaling pathway could produce IL-1*β*, TNF-*α*, IL-6, and other inflammatory cytokines. All these cytokines could bind to their corresponding receptors in microglia [37] and astrocytes [38] and enhance inflammatory responses in the brain parenchyma [35, 39]. Additionally, activated ECs upregulate nitric oxide synthase and cyclooxygenase-2 synthase to aggravate dysfunction of ECs [40–42]. The activated ECs accelerate formation of microthrombus, thereby worsening BBB permeability and exacerbating brain dysfunction [43–45]. Exploratory therapies targeting ECs could alleviate sepsis by suppressing endothelial inflammatory responses, microthrombus formation, and organ dysfunction [46, 47].

BBB destruction disrupts the relative isolation of the CNS, and various neurotoxic substances can directly damage the CNS and exacerbation of gliosis that manifest as the increased cell number and the corresponding marker proteins, Iba1 and GFAP, are stained and enhanced [48]. All of which lead to a neuroinflammatory cascade [35], which may promote sepsis-driven brain dysfunction.

### 2.3. The Complement System

As an important component of innate immune system, the complement system is involved in the homeostasis of brain [49, 50]. Many organs in the body, such as liver, kidney, and brain, could produce complement proteins [51]. In the brain, microvascular ECs, microglia, astrocyte, and even neuron are the source of complement proteins under certain conditions [51, 52]. Complement activation is a very important factor during sepsis-induced brain dysfunction and may be a potential therapeutic target [53, 54]. Complement C5a levels in cerebrovascular ECs, microglia, and deep neurons increase during sepsis [55]. C5a as a regulatory site influences cronobacter sakazakii related contextual-associated learning through NF-*κβ* and ASK1 pathways [54]. And the C5a neutralizing antibodies or inhibition of its receptor reduces organ dysfunction and BBB damage [56–58]. C3, another important member of the complement system, is also involved in BBB breakdown due to sepsis [59]. In the hippocampus of LPS-stimulated mice, the C3 secreted by astrocyte and C3a receptor (C3aR) expressed by microglia was upregulated [60]. The interaction of C3 and C3aR induced the activation of microglia [60]. Although not explored further, the authors suggested that the activated microglia may induce the loss of inhibitory synapse-related protein through disrupting the transmission of GABAergic synapses, which at last exacerbating cognitive impairment [60]. The complement system also disrupts the BBB via synthesis of inflammatory cytokines and chemokines, which causes edema and neutrophil infiltration [59, 61].

### 2.4. Neuroinflammation

#### 2.4.1. Activation Signals of Neuroinflammation

During sepsis, homeostasis is maintained via coordination of the nervous, immune, and endocrine systems [62–64]. It is known that sepsis information is transmitted from the periphery to the CNS through the following 3 pathways to regulate the neuroendocrine system, the vegetative nervous system, and behavioral response: (1) the afferent nervous system was represented by the vagus nerve [65]. Inflammatory cytokines such as IL-1*β* bind to IL-1*β* or prostaglandin E2 receptor in vagal fibers and increase vagal activity, which at last affects the nucleus tractus solitarius (NTS) by glutamate. The catecholaminergic neurons of the NTS project to different nuclei and cause sickness behaviors [66–68]. Neurotransmitters and neuromodulators derived from bacteria, gamma-aminobutyric acid, noradrenalin, serotonin, dopamine, and acetylcholine could change the state of brain by activating the vagus nerve [69]. Recently, the vagus nerve, as a bridge connecting the gut-brain axis, has been studied extensively. The gut-derived serotonin was released by gut mucosal enterochromaffin cells in response to stimuli including LPS [70, 71]. The increased serotonin by oral selective serotonin reuptake inhibitors upregulating vagal fiber activity, which at last improving depression [72]. Vagus nerve can sense bacterial metabolites (butyrate, propionate, acetate, and valerate) and serotonin through free fatty acid receptors (FFARs) and 5-HT_3_/5-HT_4_ receptors, which facilitate to transmit signals from gut to the brain [73]. And other different kinds of hormones induced by enteroendocrine cells also transmit peripheral inflammatory signals through the vagus nerve to the autonomic nervous system nucleus, neuroendocrine centers, and behavioral centers, driving corresponding changes [66, 74–76]. The vagus nerve is composed of afferent and efferent nerves, and the activated vagus nerve also acts as an anti-inflammatory mechanism: activating the hypothalamic-pituitary-adrenal axis, thereby producing endogenous steroids and suppressing inflammatory responses [77].The activated vagus nerve also releases acetylcholine, which binds to nicotinoid receptors on the surface of macrophages to suppress inflammatory responses via negative feedback [78]. Vagus activation has also been shown to suppress neuroinflammatory responses by regulating the activation of microglia [74] and the number of astrocytes in the hippocampus and dentate gyrus [79], alleviate the loss of neurons [80], and at last improve sepsis-induced brain dysfunction. Future studies should focus on drug exploration or clinical transformation [74]. (2) Peripheral inflammatory mediators and LPS may directly reach the CNS via the periventricular apparatus [65], which is located between the 3^rd^ and 4^th^ ventricles adjacent to the neuroendocrine nuclei and plant nervous system nuclei. Due to the lacking of BBB and expression of receptors associated with innate and acquired immunity, CNS can directly detect peripheral inflammatory mediators, such as TNF-*α*, IL-1*β*, and IL-6, which activates corresponding nerve nuclei, leading to behavioral changes, fever, and severe nerve damage [75, 81]. (3) Circulating inflammatory mediators enter the CNS via the damaged BBB [30]. Sepsis causes inflammatory mediators to directly access the CNS and to damage the brain parenchyma [27]. Upon entering the CNS, they activate corresponding deep nuclei by influencing neural hormone and cholinergic neurons, gamma-aminobutyric acid, beta-endorphin, and adrenocorticotropic hormone releasing function. These result in neuroendocrine, behavioral, and cognitive impairment and even affect immune regulation [82–85]. Many mediators, including cytokines, prostaglandins, and nitric oxide (NO), are involved in the activation of proinflammatory responses in the CNS by regulating neurotransmitters and neurosecretion [86]. Moreover, cholinergic and other anti-inflammatory response systems are also activated, suggesting the existence of a proinflammatory/anti-inflammatory homeostasis in the activation signal [87].

#### 2.4.2. Brain Cell Dysfunction

Sepsis produces proinflammatory cytokines that enter the brain parenchyma and cause changes in oxidative stress levels, leading to brain cell dysfunction [88]. The inflammatory cytokines that enter the brain parenchyma bind to receptors on the surface of brain cells and amplify inflammatory responses [30]. Sepsis affects various brain regions differently, with the cortex and hippocampus being highly susceptible [89]. Affected neurons may undergo apoptosis and pyroptosis cellular injury and death in the neural tissues [84, 90]. This may be one of the mechanisms underlying cognitive impairment [91]. Sepsis also disrupts mitochondrial function, which may result in the production of ROS and reactive nitrogen species (RNS) [92]. Additionally, damaged mitochondria can release DAMPs [93]. These effects can cause structural damage to the cell membrane and induce inflammation, causing neuronal apoptosis and cognitive impairment [94].

Microglia, a subgroup of macrophages in the brain, is an important component of glia and has important roles in injury and CNS disease [95]. Recently, myeloid cells in meninges are mainly derived from bone marrow of skull and vertebral, which are transported through vascular channels between the skull and the dura mater [96]. Under some circumstances, such as brain injury or neuroinflammation, these myeloid cells could migrate into parenchyma and differentiated into macrophages to modulate immune response [96]. Therefore, the microglia are constantly self-renewing through myeloid cells in skull and vertebral. In normal physiological conditions, microglia are inhibited by various multiple inhibitory factors. The cytokines TGF-*β* could induce the quiescent phenotype of microglia by Smad signaling [97]. The complete neuron-microglia connection circuits, CD200 on neuron interacts microglial CD200R, will keep the microglia in its inactivated, resting state [98]. The well-functioning DAP12-trem2 signaling pathway is also a matter of way for inhibiting microglia activation. In this state, microglia are involved in immune surveillance, synaptic modification, and neurogenesis of the surrounding environment through a large number of branches emanating from the cell [99–101]. Microglia also support neuronal survival and growth by secreting neurotrophic factors like insulin growth factor 1 (IGF1), brain-derived neurotrophic factor (BDNF), transforming growth factor *β* (TGF*β*), and nerve growth factor (NGF) [102, 103]. Sepsis relieves the inhibitory mechanisms and releases various other factors like TNF, iNOS, or glucose and confers proinflammatory function to microglia, which produce a range of proinflammatory and neurotoxic factors, thereby expanding inflammatory responses and neuronal damage in the CNS. The activated microglia are divided into two phenotypes based on antigen markers and function: M1 and M2. M1 is the classic activation of microglia, mainly expressing surface antigens CD16, CD32, and CD86 and secreting IL-1, IL-6, and TNF-*α*, which mediate inflammatory response and produce cytotoxic effects. These effects contribute to brain injury and cognitive dysfunction [104]. Activated microglia may also exacerbate brain dysfunction by altering the permeability of the BBB [105]. M2 phenotype is an alternative activation of microglia, mainly expressing antigens chitinase 3 like Protein3 (Chi3l3), arginase-1 (ARG-1), and CD206. Secretion of insulin like growth factor-1 (IGF-1) and transforming TGF-*β* and other anti-inflammatory factors can inhibit the excessive inflammatory response. M2 phenotype microglia can also secrete neurotrophic factors and play a protective role on neurons. Increasing the proportion of M2 microglia can improve the brain dysfunction in sepsis [106]. In conclusion, as the first line of defense against pathogens or injury, microglia are crucial in the maintenance of CNS homeostasis.

Astrocytes maintain CNS homeostasis through a wide range of functions, including ion homeostasis and neurotransmitter metabolism, fluid balance, regulation of local blood flow, neurogenesis, maintenance of synaptic connectivity, and plasticity [107]. Astrocytes express a wide range of receptors for DAMPs and PAMPs, including TLRs, NLRs, double-stranded RNA-dependent protein kinases, scavenger receptors, mannose receptor, complement components, and mediators like CXC chemokineligand-10 (CXCL10), chemokine (C-C motif) ligand 2 (CCL2), IL-6, and B-cell-activating factor of the TNF family (BAFF) [108]. After activation, astrocytes secrete proinflammatory factors that induce and/or regulate neuroinflammation. Astrocyte-derived factors with proinflammatory activity are represented by the following: (i) chemokines (including monocyte chemoattractant protein-1 (MCP-1/CCL2), CCL5 (RANTES), CCL7, CCL8, CCL12, CXCL1, CXCL8 (IL-8), CXCL9, IFN-*γ*-inducible protein-10 (IP-10/CXCL10), CXCL12, and CXCL16), (ii) cytokines and growth factors (including IL1-*β*, IL-6, IL-11, IL-15, IL-17, TNF-*α*, BAFF, and VEGF), (iii) intracellular signaling factors (including NF-*κ*B, SOCS3, and Act1), and (iv) small intercellular effector molecules (including PGE and NO) [109]. These proinflammatory cytokines exacerbate neuronal damage, leading to brain dysfunction in sepsis. Astrocytes also regulate the neuroinflammation during sepsis by controlling microglial activation. Microglia activation in inflamed brain triggers a pronounced neurotoxic phenotype characterized by the release of multiple cytokines and ROS/RNS, which contribute to cell death in specific vulnerable brain areas [110]. During sepsis, PAMPs could bind to the corresponding receptors on the surface of astrocytes, such as TLR4, activate the NLRP3 inflammasome to induce pyroptosis, and release histones to damage neurons [111]. By inhibiting glutamate reuse by astrocytes, sepsis affects neuronal function. Astrocytes are an important part of the BBB and control its permeability. Upon activation, astrocytes produce VEGF-A and thymidine phosphorylase (TYMP/endothelial cell growth factor 1, ECGF1), which suppress the expression of TJ proteins in cerebral ECs, thereby enhancing breakdown of the BBB [34]. In response to endotoxemia, astrocytes also secrete CCL11, which impairs learning and memory in the adult brain and triggers microglia migration and ROS production, thereby causing hippocampal neuronal damage, behavioral changes, and memory impairment [112].

Neurons can be damaged through a variety of mechanisms during sepsis. Activated microglia can cause neuronal damage by releasing inflammatory cytokines and ROS [113, 114]. Microglia can also induce the transformation of A1 astrocytes to injury neurons [115]. In addition to secreting inflammatory cytokines [116], astrocytes also affect the release of neurotransmitters, which at last leads to neuronal injury [117]. Sepsis can dysfunction the autophagy and pyroptosis homeostasis [118] and activate ferroptosis [119] and endoplasmic reticulum stress of neurons, all of which could lead to neuronal damage [120]. Abnormal activation of neuronal membrane receptors can transmit stimulus signals and induce PANoptosis [121]. Recently, the interactions of organs are getting a lot of attention. The gut can damage neurons through the accumulation of cytokines [122]. In conclusion, in sepsis, various direct or indirect factors could lead to neuronal damage and aggravate the development of cognitive impairment.

### 2.5. Microcirculation Dysfunction

Normal microcirculation is essential for maintaining CNS function. Sepsis triggers coagulation disorder by activating ECs, which enhances coagulation cascade activity and promotes microthrombus formation [43]. The activated brain endothelium activates thrombin, which regulates coagulation via prothrombin cleavage by Factor X. Thrombin then converts soluble fibrinogen to fibrin and activates platelets, resulting in microocclusions [44]. Continuous microthrombus formation exacerbates focal ischemia by occluding the vasculature beyond the initial occlusion sites. The lack of oxygen and nutrients supply further aggravates EC activation and induces coagulation dysfunction, leading to ischemia or hemorrhagic injury [123]. Moreover, basic and clinical studies show that sepsis damages the regulatory function of cerebral vasomotor and blood pressure autoregulation [124], affects cerebral perfusion, and aggravates brain injury [125]. The degree of damage to the cerebral microcirculation negatively correlated with the prognosis of sepsis-induced brain dysfunction. Microcirculatory dysfunction is characterized by rapid onset and clearly precedes changes in neurovascular coupling and systemic circulation [126]. However, there are few simple and effective methods of clinically detecting cerebral microcirculation. Although systemic circulation parameters may change with sepsis, their applicability to microcirculation is unclear. At present, evaluation and optimization of cerebral perfusion are still inconclusive, but necropsy reports have confirmed that sepsis it causes multiple microinfarcts, especially in areas with relatively low cerebral blood flow. And an MRI study revealed that patients with sepsis are at a higher risk of ischemic stroke.

In conclusion, impaired microcirculation may contribute to the pathogeneses of sepsis-induced brain injury, especially, sepsis-induced cognitive impairment. Currently, there are 2 hypotheses on sepsis-induced cognitive impairment: (a) the hypothesis that neurodegeneration involves microglial activation and (b) the hypothesis that impaired microcirculation involves blood vessels. These hypotheses are interwoven and warrant further investigation.

### 2.6. Brain Dysfunction

Although neuroinflammation usually occurs in a diffuse form, patients with sepsis usually suffer from multiple factors and some brain areas are particularly sensitive to neuroinflammation or lack BBB protection, making them more vulnerable to direct attack by peripheral inflammatory cytokines. The hippocampus is particularly vulnerable to damage during sepsis because inflammation, ischemia, hypoxia, and blood sugar disorders can all injure the hippocampus. Moreover, these changes may cooccur during sepsis. Inhibition of oxidative stress in the hippocampus may reduce injuries and cognitive impairment in sepsis [127]. Additionally, other areas of the brain, including the cortex, cerebellum, and brain stem, are also damaged by sepsis [128–130]. Thus, it is believed that sepsis-driven brain injury presents in a diffuse form and is closely associated with cognitive impairment.

Sepsis-induced brain stem dysfunction results in changes in consciousness as well as cardiovascular and immune system dysfunction and contributes to poor patient prognoses [30]. The brain stem controls immune responses through the sympathetic and parasympathetic nervous systems [131]. During sepsis, changes in cholinergic neurotransmitters are also an important driver of brain stem dysfunction. Brainstem cholinergic pathways can diminish cardiovascular and neuroinflammatory actions during endotoxemia [132]. The brain stem nucleus is susceptible to sepsis and treatment for this can alleviate sepsis-induced brain dysfunction, including neuroinflammation and cognitive dysfunction [132].

Additionally, sepsis also has driven damage of the neurotransmitter system [133], which at last promotes the incidence of brain injury [134, 135]. These neurotransmitter systems include acetylcholine, GABA, dopamine, norepinephrine, serotonin, and glutamate [136–139]. During sepsis, neurotransmitter synthesis is also altered by neurotoxic amino acids like NO, tryptophan, and phenylalanine. Metabolic dysfunction due to liver and kidney failure caused by sepsis and various drugs can affect neurotransmitter synthesis and release.

## 3. Clinical Symptoms of Sepsis-Induced Brain Dysfunction

Clinically, sepsis-induced brain dysfunction is characterized by focal neurological deficits, cognitive impairments, depression, attention decline, mood disorders, and movement-coordination problems, as well as reduced rationality, awareness, comprehension, intelligence, mental processing, and social interaction. Psychomotor agitation, anxiety syndrome, reduced visual representation (episodic and semantic memory), loss of visual acuity, executive and intellectual changes, and disturbed circadian rhythm have also been observed in cases of sepsis-induced brain dysfunction [4]. Moreover, simultaneous changes in cerebral and cardiovascular function have been reported, whereby cardiopulmonary resuscitation and artificial ventilation were used as remedies [65]. Sepsis-induced brain dysfunction also affects peripheral circulation and its link with brain parenchymal signal intensity [45]. Impaired cerebral microvasculature and decreased microcirculation, as well as overall reduction in total and perfused blood vessels and functional red blood cell capillary densities, are key features of sepsis-induced brain dysfunction. Another key feature is brain microcirculatory abnormalities during the onset and progression of sepsis induced brain dysfunction [140]. Thus, because sepsis-induced brain dysfunction lacks specific neurological indicators, clinicians should use exclusion diagnosis based on patient history to determine if brain dysfunction is due to sepsis.

## 4. Diagnosis

Diagnosis of sepsis-induced brain dysfunction is exclusionary and requires that other potential causes of neurological dysfunction, including drug effects, metabolic disorders, primary central diseases like meningitis, encephalitis, cerebrovascular diseases, and epilepsy, and noninfectious systemic inflammatory reactions like burns, severe pancreatitis, and trauma, are first excluded [141]. Neurological examination is the basic means of identifying patients with sepsis-induced brain dysfunction but is not suitable for mechanically ventilated patients in deep sedation. Electroencephalogram can be effective but should be combined with other examinations for comprehensive assessment [142, 143].

Currently, some biomarkers have been used to assess sepsis-induced brain dysfunction. But the evidence is insufficient, which has not been promoted clinically (Table 1).

## 5. Treatments

Currently, there are no specific treatments for sepsis-induced brain dysfunction and treatment mainly focusses on symptoms and may include control of sepsis and minimizing the injury to the CNS. Symptomatic treatment of sepsis-induced brain dysfunction does not differ significantly from sepsis treatment. Early resuscitation is considered a key therapeutic strategy against sepsis. Fast fluid restoration is proposed as a primary measure so as to restore hemodynamic stability and systemic oxygen delivery, which reduces neuroinflammation, stroke volume, and need for vasorepressor agents. However, this approach carries some risks, including hyperchloremic metabolic acidosis, hyperkalemia, and pathologic immune activation, as well as cellular damage, bleeding disorders, renal failure, or life-threatening allergic responses. After fluid therapy during the early resuscitation process, vasorepressor therapy that links with a normal arterial pressure can reduce the severity of sepsis.

In recent years, specific treatments for sepsis-induced brain dysfunction have been sought. Vagus nerve stimulation attenuates sepsis-induced peripheral inflammation and activates afferent nerve fibers at axonal projections. This stimulation attenuates the expression of proinflammatory cytokines in the brain. Vagus nerve stimulation also reduces sepsis-induced hypotension, disseminated intravascular coagulation, fibrinolytic activity, and systemic organ dysfunction. Free radical generation and oxidative stress contribute to the progression of sepsis-induced brain damage. Thus, antioxidant therapies have been proposed for managing sepsis-associated brain dysfunction. In response to stress, the hypothalamus secretes corticotropin releasing hormone, resulting in cortisol secretion by adrenal glands. Hence, glucocorticoids have been considered for treating sepsis-induced brain dysfunction [144]. Surrogate markers and modulators of neuroimmune axis have also been considered for treating sepsis-induced brain dysfunction. CNI-1493 is a guanylhydrazone that reduces neuroinflammation by inhibiting P38/MAPK signaling. Moreover, *α*- or *β*-adrenergic receptor modulation may also stimulate recovery from dysregulated immune responses [145]. Hypothermia has been considered a standard strategy [146]. The enzyme indoleamine 2,3-dioxygenase (IDO), which influences inflammatory processes, has been considered as a therapeutic target for treating CNS disorders. IDO inhibition not only restored adaptive immunity and energy metabolism but also improved cognitive function in sepsis [147]. The ginsenoside, Rg1, an important component of ginseng, has been reported to suppress apoptosis and autophagic degradation in the hippocampus during sepsis-induced brain dysfunction. Rg1 is reported to attenuate brain atrophy and to reduce histopathological alterations in the hippocampus. It reduces the levels of the inflammatory mediators, TNF-*α*, IL-1*β*, and IL-6, the expression of activated microglial marker, Iba1, microglia morphological changes like rounded cell bodies and shrunken neurites, macrophage infiltration, neuroinflammation, and caspase-3 activation in neurons. These Rg1-induced neuroprotective effects decreased behavioral defects in the sepsis model [148]. Treatment with recombinant club cell protein (CC16), which has anti-inflammatory and antioxidant effects, reduced sepsis-associated pathological changes in brain tissue by suppressing p38/MAPK signaling. However, neuroprotection from recombinant CCL16 involved augmented LC3II and suppressed QSTM1/p62 expression, along with the division of large autophagosomes into smaller autophagic vacuoles. This led to an increased neuronal survival due to reduced apoptosis. Depending on the protective agent, increased autophagy often protects from sepsis-induced apoptosis. A rat model of sepsis-induced brain dysfunction revealed that low-dose dexamethasone enhances autophagy, as revealed by inhibited mTOR signaling, increased LC3-II/LC3-I ratio and decreased p62/SQSTM1 in cortical neurons. Although adjunctive therapy in patients with severe sepsis-induced brain dysfunction may improve BBB dysfunction, further studies are needed to confirm this (Table 2). Despite these treatments' advances in animal, clinical applications are still to be explored.

## 6. Conclusion and Outlook

Brain dysfunction due to sepsis is often overlooked despite its high incidence and contribution to increased mortality in ICU patients. The pathophysiological mechanisms underlying sepsis-induced brain dysfunction are very complex and are mainly driven by inflammation. These processes affect brain cell metabolism by inducing oxidative stress and altering neurotransmission. The inflammatory processes mainly involve endothelial activation, impaired microcirculation, BBB defects, inflammatory mediators, and microglial cell activation. A comprehensive neurological examination is essential for diagnosing sepsis-induced brain dysfunction and should be performed daily. Active control of sepsis is the cornerstone of treating sepsis-associated brain dysfunction and should be done in a holistic framework that includes high-dose antibiotic therapy and fluid support therapy. On this basis, further research is needed to develop effective treatments for brain dysfunction. Potential future treatment strategies include vagus nerve stimulation and the regulation of neuroinflammation regulation, neuroendocrine function, and neuroimmunity.

Future research on sepsis or brain dysfunction needs to address several issues. First, quick and accurate strategies for diagnosing sepsis-induced brain dysfunction are needed. The rapidly advancing imaging techniques are getting us closer solving this problem. New neuroimaging methods that target neuroinflammation, including PET, SPECT, and new MRI protocols that have been applied in multiple sclerosis and neurodegenerative diseases may in future become applicable to sepsis. Secondly, a better understanding of pathophysiological mechanisms underlying sepsis-triggered brain dysfunction is needed. There is no doubt that the relationship between sepsis and brain dysfunction will become a major research area in the future. Currently, several treatments have been investigated using animal models, but they are all in the early stages of research and have not reached clinical trial stages. In view of its high incidence, more effective treatments are urgently needed for sepsis-induced brain dysfunction.

## Figures and Tables

**Table 1:** Suggested biomarkers to monitor sepsis-induced brain dysfunction.

Biomarkers	Significance	Location
C-reactive protein (CRP) and Procalcitonin (PCT)	Higher CRP levels indicated prolonged acute brain dysfunction [149]	Plasma
C-type natriuretic peptide (NT-proCNP)	High-peak concentration of NT-proCNP in the early phase of sepsis could predict SAE [150]	Plasma
IL-6, IL-8, IL-10, TNF-*α* and S-100*β*	Negatively associated with delirium free days [151]	Plasma
Neurofilament (Nf)	Nf could predict poorer cognitive outcome in SAE patients [151]	Cerebrospinal fluid (CSF) and plasma
Adiponectin, Tau, and neopterin	Significantly higher in patients with delirium [152]	Plasma

**Table 2:** Suggested treatments to sepsis-induced brain dysfunction.

Therapies	Mechanism
Vagus nerve stimulation	Regulation inflammatory response to protect organs [74]
Antioxidant therapies	Reduces oxidative stress to improve brain function [74]
Glucocorticoids	Regulate the secretion of hormones and anti-inflammatory [144]
CNI-1493	Regulation neuroinflammation by inhibiting P38/MAPK signaling [145]
Hypothermia	Change antibiotic pharmacokinetics [146]
IDO	Influences neuroinflammation [147]
Rg1	Anti-inflammation, suppress apoptosis, and autophagic degradation [148]
CC16	Anti-inflammatory, antioxidant, regulation autophy [153]
